# Prediction of Electrical Conductivity of Fiber-Reinforced Cement-Based Composites by Deep Neural Networks

**DOI:** 10.3390/ma12233868

**Published:** 2019-11-23

**Authors:** Dongdong Yuan, Wei Jiang, Zheng Tong, Jie Gao, Jingjing Xiao, Wanli Ye

**Affiliations:** 1 School of Highway, Chang’an University, South 2nd Ring Road Middle Section, Xi’an 710064, China; ddy@chd.edu.cn (D.Y.); yewanlis@foxmail.com (W.Y.); 2 Key Laboratory for Special Area Highway Engineering of Ministry of Education, Chang’an University, South 2nd Ring Road Middle Section, Xi’an 710064, China; 3 School of Transportation and Logistics, East China Jiaotong University, Nanchang 330013, China; highway-gaojie@st.chd.edu.cn; 4 School of Civil Engineering, Chang’an University, South 2nd Ring Road Middle Section, Xi’an 710064, China; xiaojj029@sina.com

**Keywords:** carbon fiber-reinforced cement-based composite, fiber distribution, electrical conductivity, deep learning, scanning electron microscopy

## Abstract

This study presents a deep-learning method for characterizing carbon fiber (CF) distribution and predicting electrical conductivity of CF-reinforced cement-based composites (CFRCs) using scanning electron microscopy (SEM) images. First, SEM images were collected from CFRC specimens with different CF contents. Second, a fully convolutional network (FCN) was utilized to extract carbon fiber components from the SEM images. Then, *D_SEM_* and *D_sample_* were used to evaluate the distribution of CFs. *D_SEM_* and *D_sample_* reflected the real CF distribution in an SEM observation area and a specimen, respectively. Finally, a radial basis neural network was used to predict the electrical conductivity of the CFRC specimens, and its weights (*d_i_*) were used to evaluate the effects of CF distribution on electrical conductivity. The results showed that the FCN could accurately segment CFs in SEM images with different magnifications. *D_sample_* could accurately reflect the morphological distribution of CFs in CFRC. The electrical conductivity prediction errors were less than 6.58%. In addition, *d_i_* could quantitatively evaluate the effect of CF distribution on CFRC conductivity.

## 1. Introduction

Carbon fiber (CF)-reinforced cement-based composites (CFRCs) are widely used in civil engineering (e.g., ice–snow melting pavements [1] and self-monitoring structures [2]) because of their excellent electrical conductivity [3]. Unfortunately, it is difficult to control and predict the electrical conductivity of CFRCs owing to their complex features, particularly the CF content, conductivities of cement and CFs [4], and CF distribution [5]. With the development of measurement technologies, the conductivities of cement and CFs can be calibrated with high precision. However, the real CF morphological distribution in CFRCs is varied in real-world conditions. The challenge and importance of this issue have led researchers to develop many techniques to evaluate CF distribution and predict the electrical conductivity of CFRCs.

At present, there are three approaches to evaluate CF distribution. One approach is the standard wash-out test [6,7]. As a shallow operation, this method can only indirectly characterize the distribution by measuring the content of fibers in the samples. A similar problem can also be found in the electric resistance method [8] and the microwave heating method [9]. Another approach is based on computed tomography (CT), which presents CF components in consecutive slices. Compared with the standard wash-out test, this method can characterize the real morphology of CFs, such as CF clusters. For example, Gao et al. [10] presented a grayscale clustering method to characterize CF clustering distribution using CT images with acceptable precision. Additionally, studies by Bordelon et al. [11] and Han et al. [12] presented CT-based methods for composite microstructure evaluation. However, the morphology representation capacity of CT images is limited by spatial and contrast resolution, which means that CT images can only characterize the morphology of CF bundles, not all CFs. The third approach, which may be the most promising and is the focus of this study, is scanning electron microscopy (SEM) observation. With the development of microscopy technology, SEM has been used to investigate the distribution of composites. For instance, Wang et al. [13] and Safiuddin et al. [14] used SEM to analyze the CF distribution characteristics of damaged CFRC surfaces. However, SEM only plays a role in auxiliary observation and qualitative evaluation. Jiang et al. [15] used SEM to evaluate the morphological characteristics of CF mesh in CF-reinforced phase-change materials. This method cannot accurately evaluate the overall characteristics of CF mesh due to the small observation ranges of SEM. In addition, SEM is designed to observe and evaluate the interfacial strength [16,17], ultimate tensile strength [18], and morphology of CF and cement matrixes [19]. In summary, although SEM can accurately observe the real fiber morphological distribution of CFRCs, there are still two problems remaining: (1) the small observation range of SEM makes it impossible to accurately reflect the overall fiber distribution characteristics of fiber-reinforced materials and (2) the complex background and the status of CFs in SEM images makes it difficult to extract CFs and CF bundles from images, meaning it is impossible to quantitatively evaluate fiber distribution.

A main problem of CF distribution evaluation is that it is difficult to control and predict CFRC properties, especially conductivity. Nevertheless, several methods have been proposed. The first comprises equation-based methods. For example, Wang et al. [20] proposed surface-based cohesive performance equations for predicting the surface performance of CFRC. Sun et al. [21] proposed an equation-based method for equivalent analysis and failure prediction of CFRCs. In general, these methods are limited by various applicable conditions to utilize empirical equations for predicting CFRC properties. Moreover, empirical constants are difficult to measure because some predictions are extrapolative in real-world conditions. Another approach comprises data-driven methods, such as support vector machines [22] and artificial neural networks [23]. However, the accuracy and stability of these methods sometimes make it difficult to meet the needs of current research owing to the limited capacity for representing the real CF distribution or other properties in CFRCs.

With the development of computer technology, deep learning began to be used for object segmentation and property prediction using microscopic images [24]. For example, Liu et al. [25] used a convolution neural network to locate carbon bundles and predict carbon distribution in carbon powder-modified asphalt materials. Wang et al. [26] used deep-learning techniques to achieve microscopic morphology evaluation and crack resistance prediction of poly(N-isopropylacrylamide) gel-modified cement. In addition, deep-learning techniques are widely used in object segmentation [27] and performance prediction of additional materials from SEM images and CT images [28]. Interestingly, Tong et al. [29] proposed a fully convolutional network (FCN)-based method to characterize CF distribution using SEM images. However, the quantitative relationship between CF distribution and CFRC conductivity was not mentioned in conductivity prediction. In summary, deep-learning technology can evaluate CF distribution in CFRCs and predict its electrical conductivity based on SEM images. However, there have been few studies on the direct use of SEM images as input samples to analyze CF distribution and predict electrical conductivity of CFRC based on deep-learning techniques.

In this work, an attempt was made to use two deep neural networks and SEM images to characterize CF distribution and predict CFRC conductivity. There are two advantages in the proposed method: (a) real CF morphological distribution is considered to predict electrical conductivity of CFRC, which improves the prediction precision; (b) the contributions of different CFRC regions with different CF distributions to the electrical conductivity are measured quantificationally, which can be used for establishing a quantitative relationship between CFRC conductivity and carbon fiber distribution.

## 2. Sample Preparation and Property Measurement

### 2.1. Preparation of CFRCs

#### 2.1.1. Raw Materials

Three kinds of raw materials were used for the CFRC specimen: ordinary Portland cement, polyacrylonitrile-based short-cut CFs, and mixing water. Table 1 and Table 2 present the properties of cement and CFs used in this study, respectively.

#### 2.1.2. Specimen Preparation

To prepare CFRC specimens with different electrical conductivities, four CF contents were designed separately as 1‰, 3‰, 5‰, and 8‰ of cement mass. The water/cement ratio was 0.33 in all CFRC specimens. The after-mixing method was used to prepare the specimens [30]. First, ordinary Portland cement and mixing water were stirred by a cement mortar mixing at 60 rounds/min. The stirring time was 30 s. Second, CFs, sodium carboxymethyl cellulose, and tributyl phosphate were added into the mixture. The mixing time was 120 s. Finally, the mixture was infused into 40 mm × 40 mm × 160 mm models and cured for 28 d in conditions where the temperature and relative humidity were controlled at 20 ± 2 °C and 93%, respectively. In total, 24 CFRC specimens with four CF contents were prepared.

### 2.2. Electrical Conductivity Measurement

**Figure 1:**
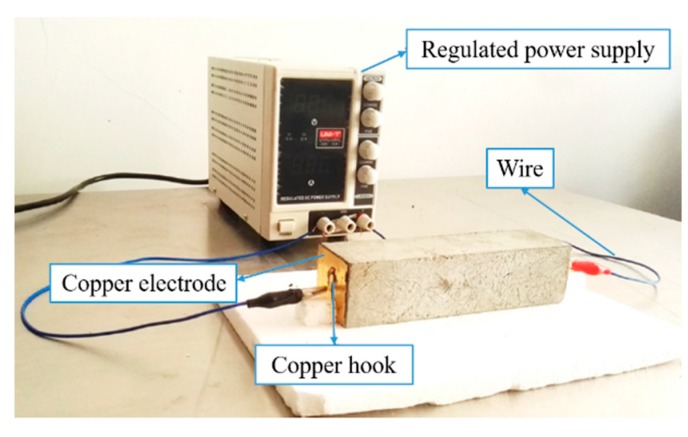
Electrical conductivity measurement device.

The CFRC conductivity was measured by a piece of custom-designed device, as shown in Figure 1 [10]. Two copper sheets, each with a sizes of 37 mm × 37 mm × 0.6 mm, were bonded to each CFRC specimen as electrodes. The copper sheets were connected with a voltage regulator through a conductive wire, and the voltage was held constant at 30 V during the measurement. The electrical conductivity of specimens was obtained by

(1) $$\rho =\frac{Rs}{L}$$

where *ρ* is the electrical conductivity (S/m); *R* is the resistance of a CFRC specimen (Ω); *s* and *L* stand for the conductive area and length, which were 37 mm × 37 mm and 160 mm, respectively, in this study.

### 2.3. SEM Image Acquisition

**Figure 2:**
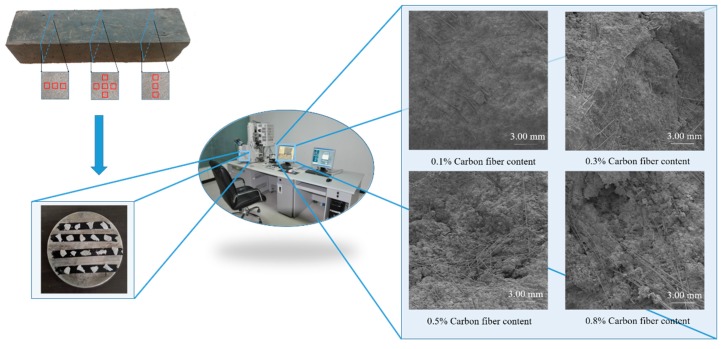
Observation areas of carbon fiber (CF)-reinforced cement-based composites (CFRC) and parts of the SEM images.

The specimens were observed by SEM. The technical parameters used were 3.5 nm resolution under high vacuum mode and 2 μA maximum beam. The samples were coated with gold in a sputter coater. The observation positions and selected results are presented in Figure 2. In total, 960 SEM images with a resolution of 1280 pixels × 1024 pixels were collected. To conduct image segmentation, dispersion evaluation, and conductivity prediction using deep neural networks, the original images were cropped into 19,200 images with a resolution of 256 pixels × 256 pixels. The CF areas in each cropped image were labeled manually. The cropped images and labels were used as the database for the deep neural networks.

## 3. Evaluation and Prediction Method Based on Deep Learning

In this study, two deep neural networks were developed for predicting the electrical conductivity of CFRCs. An FCN model was utilized to segment different components from SEM observation areas, and the results were used to calculate the CF distribution (Section 3.1). Then, a radial basis neural network (RBNN) was designed to predict the electrical conductivity of CFRCs and evaluate the effect of CF distribution on electrical conductivity (Section 3.2).

### 3.1. Evaluation Method for CF Distribution

#### 3.1.1. CF Segmentation

**Figure 3:**
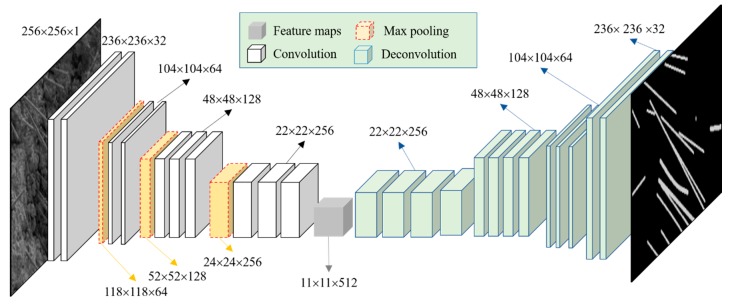
Structure and parameters of fully convolutional network.

In this study, an FCN model was used to segment different components (e.g., CFs) from SEM images. The model structure and parameters are shown in Figure 3. The FCN model was mainly composed of three parts: convolutional layers, pooling layers, and deconvolution layers.

The convolutional layer in the FCN was utilized to obtain task-related features of input images or data. A convolution operation can be summed as

(2a) $$x^{c}=ReLU\left(x^{c-1}\times k_{i}^{c}+b_{i}^{c}\right)$$

(2b) $$ReLU\left(x\right)=\{\begin{array}{cc} x & x>0\\ 0 & x\leq 0 \end{array}$$

where **x***^c^*^−1^ and *x^c^* are the input and output data for the *c*^th^ convolutional layer, respectively; **k***^c^_i_* and *b^c^* stand for the weights and bias in the *i*^th^ kernel, respectively.

As another key layer in the FCN, the pooling layer was utilized to reduce the size of the input data to avoid overfitting. Pooling was utilized in this research as follows:

(3a) $$z^{p}=\max\left(x^{p-1}\right)+b^{p}$$

(3b) $$x^{p}=\frac{1}{1+e^{z^{p}}}$$

where **x***^p^*^−1^ and *x^p^* are the input and output data for the *p*^th^ convolutional layer, respectively; max() is the maximum operation.

The deconvolution layer conducted several deconvolution operations based on the calculation results of the convolution layer and pooling layer to segment the CFs in a SEM image. The processes of deconvolution are summarized as

(4) $$y_{u+1}^{i}=\sum_{k-1}^{K}z_{u,k}^{i}⊕f_{k,c}^{u}$$

where $f_{k,c}^{u}$ denotes deconvolution weight matrix for the *u*^th^ deconvolutional layer, and ⊕ represents a deconvolutional operation. Details of a deconvolution operation are presented in [31]. The deconvolution operation is based on the convolution operation and carries out complement operation.

In this study, the aforementioned database with 19,200 SEM images was used to train the FCN; 11,520 and 3840 images were randomly selected to train and validate the FCN, respectively, while the remaining 3840 images were used to test it. The FCN model was trained using a forward-feedback algorithm to adjust the parameters of *k^l^_rot_*, *b^l^*, *b^l^*^+1^, and *f* to accurately segment CFs. The learning rate of the training FCN model was 1 × 10^−4^, and 200 SEM images of training samples were used for every 50 iterations. The effect of each iteration was evaluated using pixel-by-pixel cross-entropy loss. The number of training iterations was 18,000. After every 200 iterations, validation samples were utilized for verification, and the verification results were reviewed by pixel-by-pixel cross-entropy loss. After training, the test samples were used for verification, and the test results were evaluated using *Precision*, *Recall*, and *F-Measure* [29]. The three indexes are expressed as

(5a) $$Precision=\frac{TP}{TP+FP}$$

(5b) $$Recall=\frac{TP}{TP+FN}$$

(5c) $$F-Measure=\frac{2\times Precision\times Recall}{Precision+Recall}$$

where *TP* and *TN* are the true pixels of CFs and backgrounds in a SEM image, respectively, while *FP* and *FN* are the wrong pixels of CFs and backgrounds, respectively.

#### 3.1.2. Evaluation of CF Distribution

In this study, the following two indexes were proposed to evaluate the CF distribution:

(6a) $$D_{SEM}=\frac{A_{no}+A_{single}}{A_{no}+A_{cluster}+A_{single}}$$

(6b) $$D_{Sample}=\frac{1}{n}\sum_{i=1}^{n}D_{SEM,i},$$

where *A_single_*, *A_cluster_*, and *A_no_* are the CFs, clusters, and non-CF parts in a SEM image, respectively; *n* is the number of SEM images used to calculate *D_Sample_*. As the segmentation results reflect the real morphological and distribution characteristics of CFs in CFRCs, the two indexes can be regarded as the real results of CF distribution in certain observation areas. Compared with traditional CF distribution evaluation methods, this method is more real and accurate.

### 3.2. Electrical Conductivity Prediction Method

**Figure 4:**
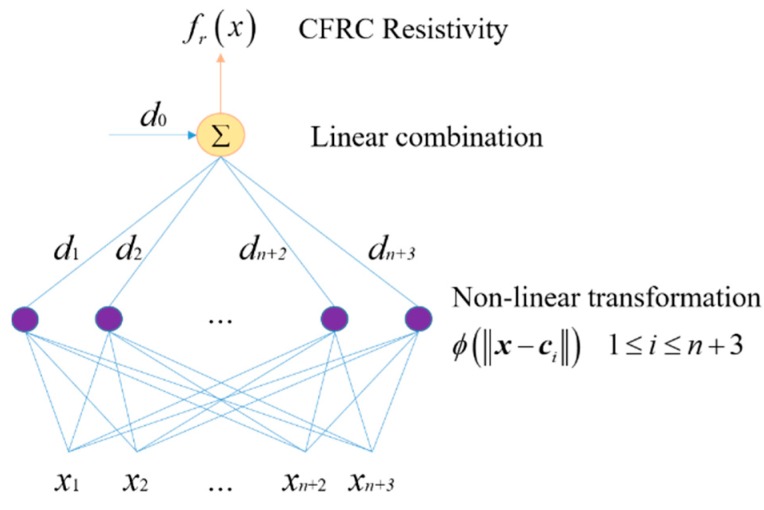
Structure and parameters of radial basis neural network.

In this study, a RBNN model was used to predict the electrical conductivity of CFRCs and evaluate the effect of CF distribution on electrical conductivity. The structure and parameters of RBNN are depicted in Figure 4. As shown in the figure, there were *n* + 3 input data.

Existing studies [32,33] have demonstrated that the electrical conductivity of CFRC is mainly related to the electrical conductivity, mass fraction, and distribution of CF. Therefore, *x*_1_ and *x*_2_ in the RBNN were the electrical conductivity and mass fraction of CFs (shown in Table 2), respectively, while *x*_3_ was the electrical conductivity of cement (shown in Table 1). The parameters *x*_4_ − *x*_n+3_ were used to characterize the CF distribution. To fully consider the effect of different CF distributions in different regions of CFRCs on electrical conductivity, the segmentation results of the FCN were directly utilized to characterize the distribution of CFs. Thus, *x*_4_ − *x*_n+3_ were the segmentation results. From the point of view of deep learning, as the results of the FCN are directly used for the RBNN, the CF distribution in each SEM image can be regarded as a low-level feature to form the conductivity property of a CFRC, which is a high-level feature. Thus, the RBNN using SEM segmentation results can still be regarded as a deep neural network because it maps learned low-level features to a high-level feature [34].

In this study, 24 CFRC specimens with 0.1%, 0.3%, 0.5%, and 0.8% CFRC were prepared. Five specimens from each CFRC with certain CF content were selected as training samples, and the remaining one was used as a test sample.

Existing studies have shown that *d_i_* in the RBNN can be used to characterize the contribution weight of the *i*^th^ input parameter to the final output [35]. In the RBNN, *x*_4_ − *x*_n+3_ were the results of FCN segmentation. As the real CF distribution in a certain observation area of a CFRC can be reflected by a segmentation result, *d_i_* (*i* = 4, …, *n* + 3) can be used to quantitatively evaluate the effect of CF distribution in a certain observation area on the electrical conductivity of a CFRC.

## 4. Results and Discussion

### 4.1. Performance of Evaluating CF Distribution

#### 4.1.1. CF Segmentation Results

The FCN was trained as introduced in Section 2.1. Table 3 presents the performance of FCN segmentation; only the testing performance results are reported in the table. As can be seen, the average *Precision*, *Recall*, and *F-Measure* of the FCN were 0.956, 0.927, and 0.938, respectively. This indicates that the FCN could accurately segment CFs in SEM images. Compared with the three indexes of clusters, the three indexes of dispersive CFs are more desirable. In addition, the FCN accurately segmented SEM images under different magnifications, demonstrating that the FCN model exhibited good stability on SEM magnifications. This is because the FCN with convolution and pooling layers could extract features with no influence from different backgrounds. The extracted features of CFs and clusters under different magnifications were similar, such as morphologies and grayscales. In addition, the FCN model had good robustness to size distortion.

**Figure 5:**
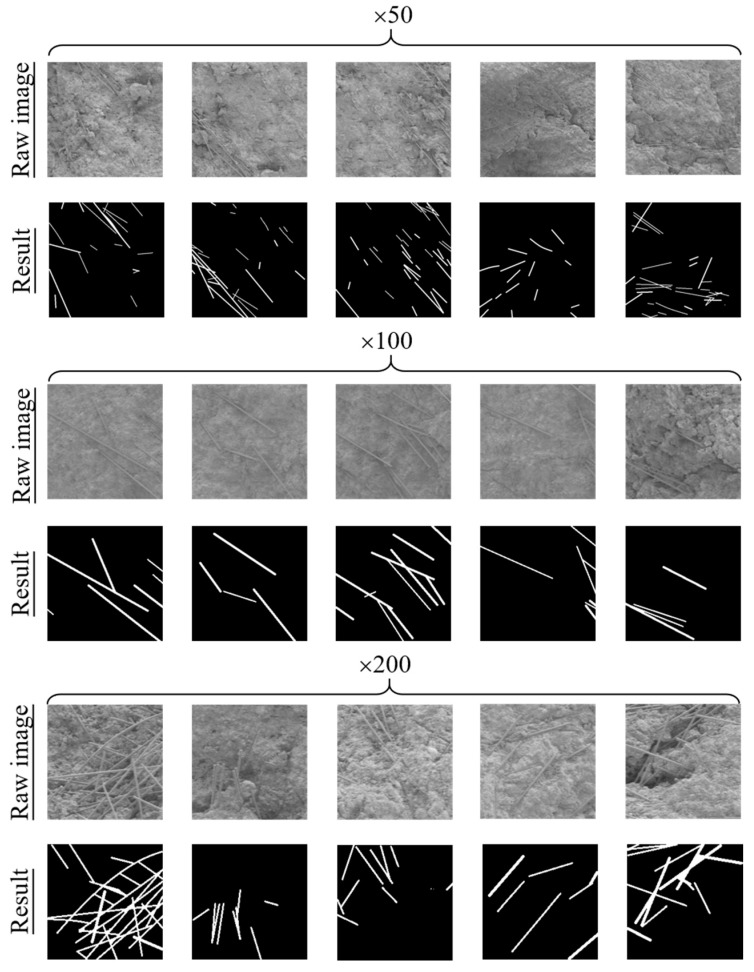
Examples of SEM image segmentation.

Several SEM images in three magnifications (50×, 100×, and 200×) and their segmentation results are shown in Figure 5. The backgrounds (e.g., cement hydration products) are black, and the fibers and clusters are white. The segmentation results showed the real CF morphological distribution in certain regions, such as CF number, CF shapes, and space distribution. Thus, the segmentation results could be used for characterization of CF distribution in the CFRC specimens. In addition, Figure 5 shows that the FCN had the capacity to segment CFs from the background (e.g., Ca(OH)_2_, hydrated calcium silicate gel, and voids), although small areas of the CF clusters were recognized as background. The incorrect recognition is the reason for the low average *Precision*, *Recall*, and *F-Measure*. In general, it had limited effect on the overall SEM segmentation. It can be seen that the FCN satisfies the accuracy and stability requirements of SEM image segmentation.

#### 4.1.2. Distribution Evaluation Results

Some studies [10,36] have proven that the tiny observation area of SEM limits its material characterization capacity. Therefore, it is necessary to increase the coverage areas of SEM images to conduct the evaluation of material properties based on SEM images. The augmented areas are more representative than the original areas of each SEM image. In addition, the testing results indicated that the FCN could segment different components in 0.18 s/image, illustrating that the FCN can conduct real-time segmentation of SEM images. Furthermore, it was able to be utilized to characterize the CF distribution in the augmented areas.

**Figure 6:**
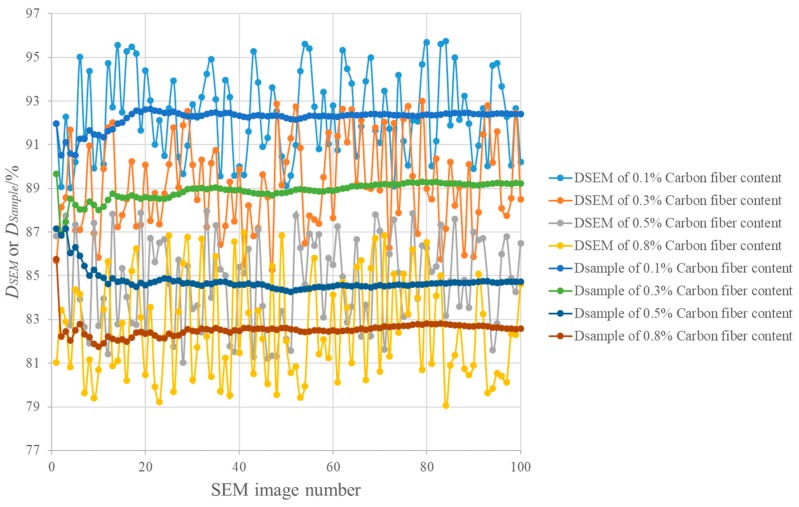
Continuous observation and CF distribution evaluation under 100× magnification.

**Figure 7:**
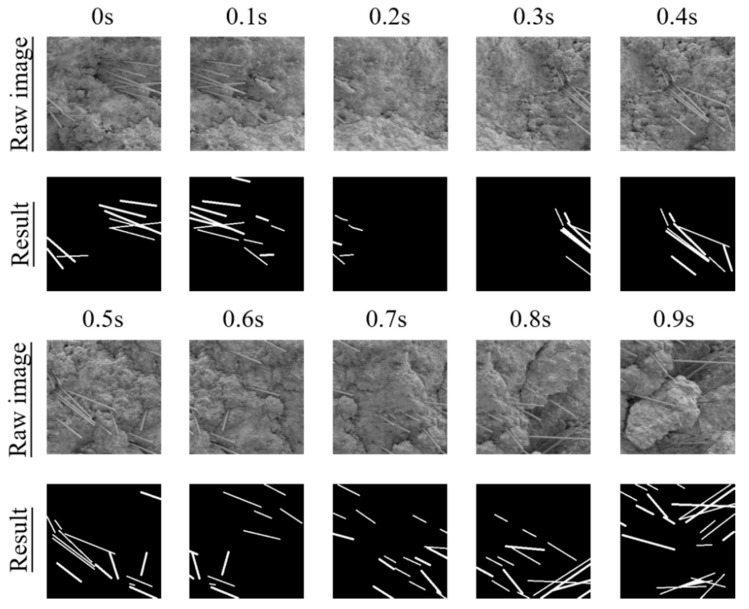
Examples of continuous observation.

Figure 6 presents the results of *D_SEM_* and *D_Sample_* for CFRC specimens with four different fiber contents. Figure 7 shows several results of real-time segmentation. Figure 6 and Figure 7 demonstrate that there were obvious differences in *D_SEM_* in different parts of CFRC specimens. For example, the maximum and the minimum *D_SEM_* values in the CFRC specimen with 0.1% CF content were 86.8% and 79.0%, respectively. The *D_SEM_* values of several regions of the CFRC specimen with 0.1% fiber content were larger than those of the CFRC specimen with 0.8% fiber content. The differences in the local CF distribution might affect the overall conductivities of CFRC specimens with different fiber contents, which were not considered in the existing studies (e.g., Gao et al. [10]). It can be concluded that a single SEM image and its *D_SEM_* value cannot be used to accurately characterize the CF distribution of CFRC specimens. Figure 6 and Figure 7 also demonstrate that the *D_Sample_* results became stable with increasing number of SEM images. This is because *D_SEM_* became more representative as the observation areas of CFRC specimens increased with increase in the number of SEM images. Thus, it is desirable to utilize *D_Sample_* with at least 80 images to characterize distribution under 100× magnification. The values of other magnification levels for *D_Sample_* can also be determined by the same method. In addition, the CF distribution of CFRC specimens deteriorated with increasing CF content under the same conditions (e.g., mixing time and method). This is because a large number of flocculations emerged as the CFs collided with each other once the fiber content increased. Both phenomena show that the continuous observation and their *D_Sample_* values were able to accurately characterize the real distribution of CFs inside CFRC specimens. Figure 6 also shows that the CF distribution decreased sharply with increasing CF content. Thus, the increase in CF content may have a negative effect on the performance of CFRC specimens. The potential reason for this is that more and more clusters emerge with the increase in CF content. Fiber clusters have some negative influence on CFRC properties, such as stress concentration [5].

### 4.2. Analysis of Electrical Conductivity Prediction Results

#### 4.2.1. Electrical Conductivity Prediction

**Figure 8:**
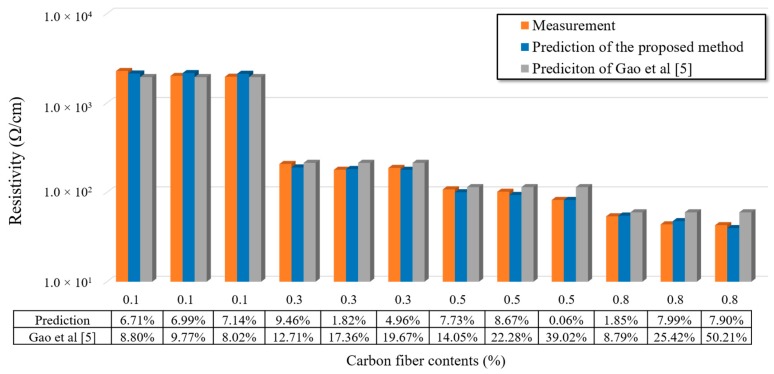
Predicted and measured electrical conductivity of specimens with different carbon fiber distribution.

Figure 8 presents the predicted and measured electrical conductivities of specimens with different CF distributions. It was found that the average error between the predicted and measured results was 6.58%, which indicates that the RBNN can accurately predict the electrical conductivities of CFRC specimens based on the electrical conductivity and distribution of CFs. This method fully considers the influence of CF distribution (CF number, CF shapes, and space distribution) on the predicted results. For example, the *D_Sample_* values of three CFRC specimens with 0.8% CF content were 83.25%, 81.69%, and 78.61%, respectively, and their predicted electrical conductivities were 53.46, 52.28 and 44.87 Ω/cm, respectively. The forecast errors were 1.85%, 7.99%, and 7.90%, respectively. Compared with the traditional methods that only consider the effects of CF content, the proposed method based on the real CF morphological distribution is more accurate and desirable. In addition, the measured and predicted results shown in Figure 8 indicate that the CF distribution affected the electrical conductivities of CFRC specimens. Although the CF content was the same in the CFRC specimens, the one with the acceptable CF distribution had the desired electrical conductivity. In addition, with increasing CF content, the CF distribution had significant effects on the electrical conductivity of CFRC specimens. Therefore, to guarantee the electrical conductivity of CFRC specimens, a proper mixing method is required to ensure satisfactory CF distribution in CFRC specimens. Figure 8 also presents the predicted results based on the method proposed by Gao et al. [5]. As Gao’s method was only based on the fiber content, the predictions were the same when the fiber content of different specimens were the same. This is inconsistent with the real conditions. In addition, the errors of Gao’s method were larger than the ones of the proposed method. Thus, the comparison indicates that it is necessary to consider the real fiber distribution to predict the properties of CFRC.

#### 4.2.2. Distribution Impact Assessment

**Figure 9:**
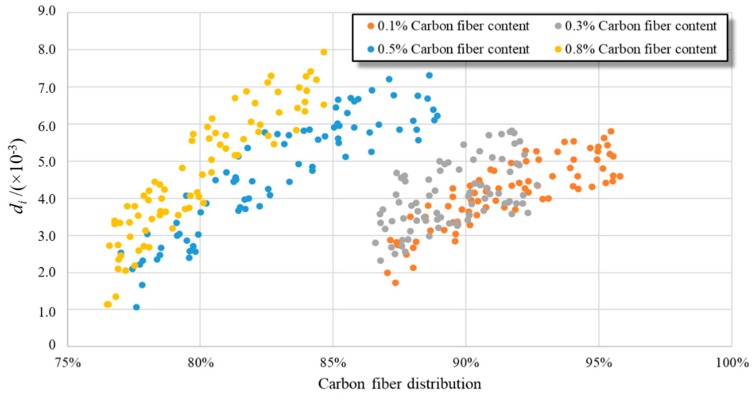
*d_i_* for the electrical conductivity prediction.

Figure 9 presents several *d_i_* values of the #2 CFRC specimens with 0.1%, 0.3%, 0.5%, and 0.8% CF content. Generally, *d_i_* increased with increasing *D_SEM_*, indicating that the contribution of a small CF distribution is less than the contribution of a large one, which is coincident with the definition of *d_i_* in the RBNN [35]. Thus, Figure 8 shows that the increase in the local CF distribution of a CFRC specimen is useful in improving the electrical conductivity of CFRC specimens. Additionally, as shown in Figure 9, the *d_i_* value can quantitatively represent the influence weights of the local distributions on the CFRC conductivity. For example, the *D_SEM_* value of the #25 SEM image observation area in CFRC specimens with 0.5% CF content had a weighted effect on the electrical conductivity of CFRC of 6.81 × 10^−3^. In addition, *d_i_* increased sharply when *D_SEM_* was less than the specific value of 81.52%, while it increased slowly when *D_SEM_* was larger than the specific value of 81.52%. For example, *d_i_* from #2 CFRC specimens with 0.5% CF content increased sharply when *D_SEM_* was less than 81.52%, while it increased slowly when *D_SEM_* was larger than 81.52%. This indicates that it is necessary to control the CF distribution in the CFRC to guarantee its electrical conductivity. This was not fully considered in previous studies as they used CF masses as the representation of the CF distribution for CFRC specimen.

## 5. Conclusions

In this study, a deep-learning method was used to characterize CF distribution and predict the electrical conductivity of CFRCs. The following conclusions are drawn:

(1) The proposed FCN was able to be utilized for real-time CF segmentation in SEM images with *Precision*, *Recall*, and *F-Measure* values of 0.956, 0.927, and 0.938, respectively. The outputs of the FCN can be used to evaluate the CF distribution in the local regions.
(2) The proposed index *D_Sample_* can be used for characterizing the real CF distribution in CFRC based on the outputs of the FCN. The *D_Sample_* results became stable with increasing number of SEM images. It is desirable to utilize *D_Sample_* with 80 SEM images for evaluating the overall distribution under 100× magnification. The values of other magnification levels for *D_Sample_* can also be determined by the same method. The CF distribution decreased sharply with increasing CF content. Thus, the increase in the CF content has a negative effect on the performance of CFRC specimens if the preparation method is not suitable.
(3) The average error between the predicted results of the RBNN and measured results was 6.58%, indicating that the RBNN was able to accurately predict the electrical conductivities of the CFRC specimens. It fully considered the influence of CF distribution (CF number, CF shapes, and space distribution) on the predicted results. The predicted results also showed that, with increasing CF content, the CF distribution had significant effects on the electrical conductivity of CFRC specimens. Therefore, to guarantee the electrical conductivity of CFRC specimens, a proper mixing method is required to ensure satisfactory CF distribution in CFRC specimens. In addition, this study only presents an example to predict the electrical conductivities of CFRCs based on their real microstructure. A generalized method should be developed in the future for predicting all other properties of CFRC.
(4) The relationship between *d_i_* and *D_SEM_* indicates that the contribution of a small CF distribution is less than the contribution of a large one. An increase in the local CF distribution of a CFRC specimen is useful in the improvement of CFRC conductivity. Additionally, *d_i_* values were able to quantitatively represent the influence weights of the local distributions on CFRC conductivity. This indicates that it is necessary to control the CF distribution in CFRCs to guarantee their electrical conductivity, rather than only controlling CF mass.

## Figures and Tables

**Table 1:** Cement properties.

Fineness (m^2^/kg)	Density (kg/m^3^)	Electrical Conductivity (Ω⋅m)	Flexural/Compressive Strength (MPa)	
			3 d	28 d
320	3114	0.72	5.9/19.5	7.2/54.1

**Table 2:** Carbon fiber properties.

Radius (μm)	Lengths (mm)	Carbon Content (%)	Elasticity Modulus (GPa)	Ultimate Tensile Strength (MPa)	Electrical Conductivity (10^−3^ Ω·cm)
4.0	2–5	95.3	241	3880	0.784

**Table 3:** Testing results of fully convolutional network (unit: %).

Category	50×			100×			200×		
	*Recall*	*Precision*	*F-Measure*	*Recall*	*Precision*	*F-Measure*	*Recall*	*Precision*	*F-Measure*
CF	0.947	0.981	0.962	0.934	0.978	0.957	0.966	0.952	0.956
CF clusters	0.908	0.976	0.934	0.892	0.971	0.938	0.834	0.977	0.902
Overall	0.925	0.976	0.950	0.914	0.975	0.943	0.902	0.961	0.929

## Data Availability

All the developed software in this manuscript are available in Onedrive via https://1drv.ms/u/s!AvKEffVDI1No06tyZjlLHMwtSEOj2A?e=s0816R. The developed software should be compiled in Caffe [37]. Access to any other materials can be requested by writing to the corresponding authors.
